# EphB1 Receptor Blockade Augments Morphine Antinociception via Inhibiting GRK2‐Mediated µ‐Opioid Receptor Internalization

**DOI:** 10.1002/advs.78099

**Published:** 2026-09-30

**Authors:** Bo Peng, Xiaqing Lin, Hongyu Yao, Cheng Li, Huabao Liao, Yuexin Liu, Boxu Cao, Xue‑Jun Song

**Affiliations:** ^1^ Department of Medical Neuroscience School of Medicine Southern University of Science and Technology Shenzhen China; ^2^ SUSTech Center for Pain Medicine School of Medicine Southern University of Science and Technology Shenzhen China

**Keywords:** µ‐opioid receptor trafficking, dorsal root ganglion, EphB1 receptor, GRK2, phosphorylation, spinal cord

## Abstract

Opioids are the primary treatment for severe pain in clinical practice. However, repeated exposure to opioids leads to tolerance, and underlying mechanisms remain elusive. The present study demonstrates that EphB1 receptors play an important role in opioid antinociception through orchestrating µ‐opioid receptor (MOR) trafficking. Morphine administration activates EphB1 receptors in the dorsal root ganglion (DRG) and the spinal cord. Deletion of EphB1 receptors in DRG neurons or vesicular glutamate transporter 2 (Vglut2)‐expressing neurons in the spinal dorsal horn (DH) augments morphine antinociception. MOR and EphB1 receptors are co‐expressed in the somata and axons of DRG neurons and DH Vglut2^+^ neurons. Deletion of EphB1 receptors in DRG neurons increases opioid‐induced suppression of calcium currents, and ablation of EphB1 receptors in Vglut2^+^ neurons enhances opioid‐induced inhibition of excitatory synaptic transmission and induction of outward currents in Vglut2^+^ DH neurons. Mechanistically, EphB1 receptors form a complex with MOR and G protein‐coupled receptor kinase 2 (GRK2) and regulate GRK2‐mediated phosphorylation of MOR at Ser^375^, thus controlling MOR internalization. These findings support the idea that activation of EphB1 receptors may facilitate the development of opioid tolerance through regulating MOR.

## Graphical abstract

Administration of opioids activates ephrinB2‐EphB1 receptor signaling. Activated EphB1 receptors induce GRK2‐mediated phosphorylation of MOR at Ser^375^, which initiates the internalization of MOR and reduces opioid antinociception at the behavioral level.

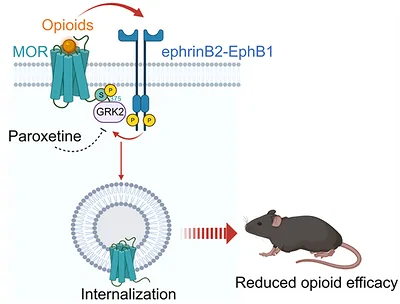

## 1 Introduction

Opioid analgesics, such as morphine, are widely regarded as the gold standard for managing moderate‐to‐severe chronic pain. However, prolonged use of these agents frequently leads to the development of tolerance, which diminishes their analgesic efficacy over time. This, in turn, often necessitates escalation and increases the risk of adverse effects and overdose [1, 2, 3]. Consequently, patients are left with limited therapeutic options, contributing to increased morbidity and mortality [4]. Therefore, elucidating the mechanisms underlying opioid tolerance is essential for optimizing their clinical effectiveness in pain management.

Opioid analgesics produce analgesic effects via µ‐opioid receptors (MO) in both the peripheral and central nervous system [5, 6], yet these same receptors are also responsible for their adverse effects [7]. MOR activation initiates desensitization through a cascade involving G protein‐coupled receptor kinases (GRKs)‐mediated phosphorylation, β‐arrestin recruitment, and subsequent receptor internalization and downregulation, ultimately leading to behavioral tolerance [8, 9, 10, 11]. Despite considerable advances in our understanding of opioid tolerance mechanisms, the precise factors through which MORs orchestrate this negative feedback regulation remain unclear.

Eph receptors, including A‐subclass (EphA1–EphA8) and B subclass (EphB1–EphB4, EphB6), constitute the largest subfamily of receptor tyrosine kinases (RTKs) [12]. Eph receptors and their ligands, ephrins, have been shown to play vital roles in synapse formation and axon guidance during the development of the nervous system [13, 14]. Studies have shown that blocking EphB receptors suppresses morphine withdrawal response and associated neuronal activation in the spinal cord [15, 16]. Our previous studies have demonstrated that prolonged use with morphine induces activation of EphB1 receptors in the spinal cord; pharmacological or genetic deletion of EphB1 receptors rescues the analgesic effect of morphine [17]. These results indicate the vital role of EphB1 receptors in regulating opioid response, however, the underlying mechanisms remain elusive.

In this study, we report that intrathecal administration of morphine induces activation of EphB1 receptors in the dorsal root ganglion (DRG) and the spinal cord. Deletion of EphB1 receptors in DRG neurons or in vesicular glutamate transporter 2 (Vglut2)‐expressing excitatory neurons in the spinal dorsal horn (DH) enhances morphine antinociception at the behavioral level and augments MOR function in DRG neurons, axon terminals, and Vglut2^+^ DH neurons in mice. Mechanistically, EphB1 receptors colocalize and interact with MOR and GRK2 in DRGs and the DH, and modulate MOR trafficking via regulating GRK2‐mediated phosphorylation of MOR at Ser^375^. Our study reveals the molecular mechanism of how EphB1 receptors regulate opioid antinociception and provides a potential therapeutic target for the treatment of opioid tolerance.

## 2 Results

### 2.1 Activation of EphB1 Receptor Signaling Impairs Morphine Antinociception

Studies have demonstrated that the ephrinB‐EphB signaling contributes to neuropathic and bone cancer pain through modulation of DRG neuronal excitability [18, 19], the DH neural excitability and synaptic plasticity, and glial cell activation [17, 18, 20]. Here, our results showed that acute morphine administration (2 nmol, i.t.) significantly increased phosphorylation of EphB1 receptors and expression of their ligand ephrinB2, but not the protein expression of EphB1 receptors in DRGs (Figure 1A). However, in the spinal cord, morphine administration increased the expression and phosphorylation of EphB1 receptors, but did not alter the protein expression of ephrinB2 (Figure 1B). These results suggest the contribution of EphB1 receptor signaling in morphine antinociception.

**FIGURE 1:**
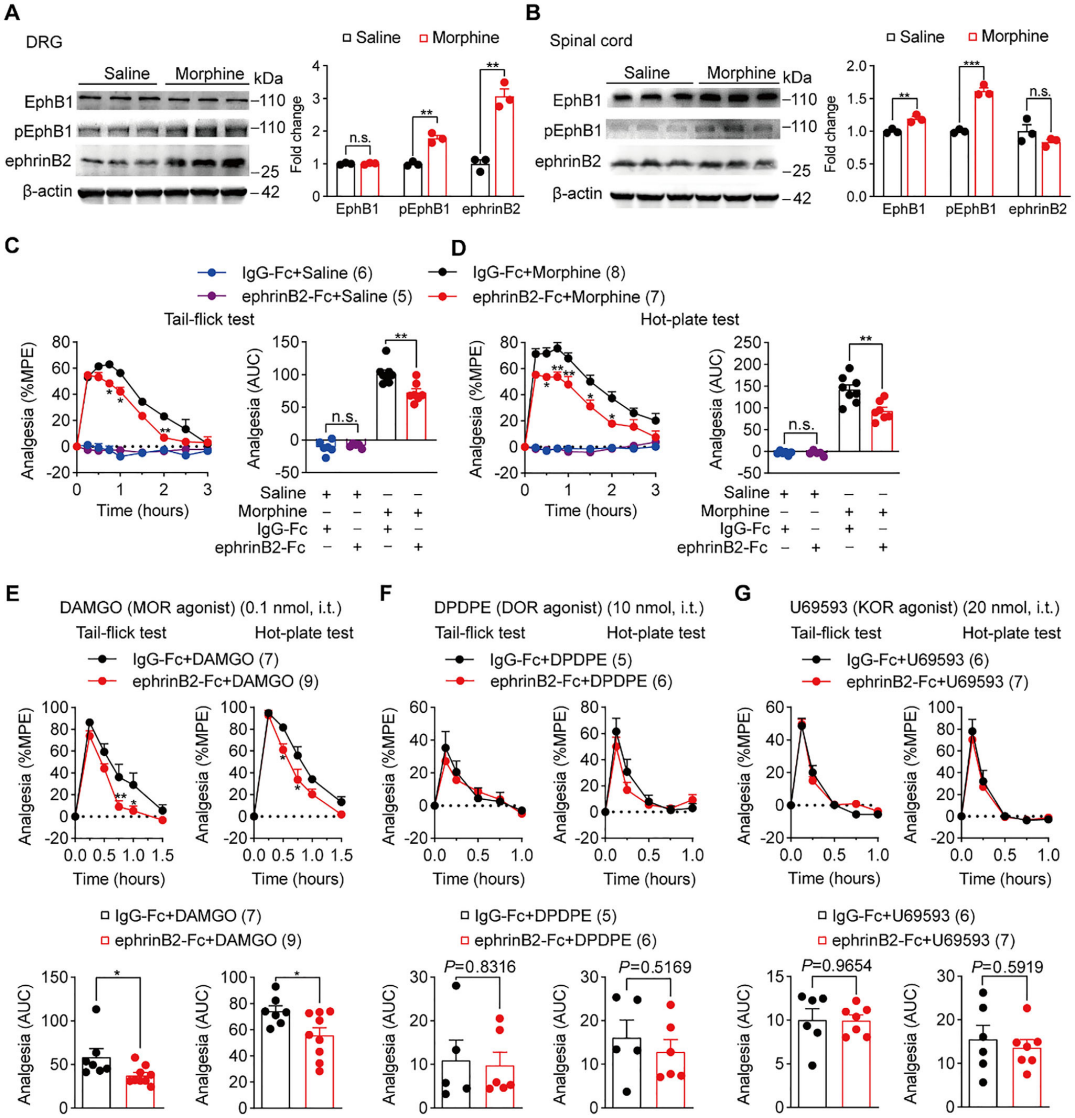
Spinal administration of ephrinB2‐Fc diminishes morphine antinociception. (A, B) Western blot showing that morphine activated ephrinB2‐EphB1 receptor signaling in DRGs (A) and in the spinal cord (B). Left, representative bands. Right, statistical summary. *n* = 3 in each group. (C, D) Intrathecal administration of ephrinB2‐Fc diminished the analgesic effect of morphine in the tail‐flick test (C) and hot‐plate test (D). Control IgG‐Fc or ephrinB2‐Fc was intrathecally injected 30 min prior to intrathecal injection of morphine. Left, %MPE. Right, AUC. (E–G) Intrathecal administration of ephrinB2‐Fc diminished the analgesic effects of DAMGO (E), but not DPDPE (F), or U69593 (G), in the tail‐flick test (left) and hot‐plate test (right). Control IgG‐Fc and eprhinB2‐Fc were injected intrathecally 30 min before intrathecal administration of DAMGO, DPDPE, or U69593. Upper, %MPE. Lower, AUC. (C–G) Sample sizes were indicated in brackets. Statistical analysis by unpaired Student's *t* test in (A) to (G), or two‐way ANOVA with Bonferroni's multiple comparison test in (C) to (G). n.s., no significance, ^*^ *p *< 0.05, ^**^ *p *< 0.01, and ^***^ *p *< 0.001.

We further examined whether the presence of EphB1 receptors is necessary for morphine antinociception. Intrathecal administration of exogenous EphB1 receptor activator ephrinB2‐Fc at a low dose (0.05 µg in 5 µL), which did not change the threshold of painful stimulation, significantly impaired the analgesic effect of intrathecal morphine administration (Figure 1C,D). Opioids produce antinociception through the µ, δ, and κ opioid receptors (MORs, DORs, and KORs, respectively). Our results showed that intrathecal injection of the specific agonists of these subtype receptors, DAMGO (MOR), DPDPE (DOR), and U69593 (KOR), elicited significant analgesic effects in naïve mice. Intrathecal administration of ephrinB2‐Fc inhibited DAMGO‐, but not DPDPE‐ or U69593‐, induced analgesic effects (Figure 1E–G). These results demonstrate that EphB1 receptors can modulate morphine antinociception via regulating MOR‐mediated antinociception, suggesting a functional interaction between EphB1 receptors and MORs.

We further investigated the mechanism by which EphB1 receptor signaling was activated following morphine administration. As previously reported, MORs are predominantly expressed by transient receptor potential vanilloid 1 (TRPV1) positive nociceptors in DRG neurons [21, 22]. Western blot analysis showed that conditional knockout of MORs in TRPV1^+^ DRG neurons inhibited the morphine‐induced increase of ephrinB2 in DRGs, demonstrating that the increase in ephrinB2 protein levels depends on MORs in DRGs (Figure S1A). However, an RNA scope assay showed a low degree of colocalization of ephrinB2 mRNA (Messenger ribonucleic acid) (*Efnb2*) and MOR mRNAs (*Oprm1*) in DRG neurons. Quantitative analysis revealed that only 8.68% *Efnb2* ^+^ neurons and 7.03% *Oprm1* ^+^ neurons expressed both mRNA, indicating that ephrinB2 was not expressed in MOR^+^ DRG neurons (Figure S1B). Also, RNA scope assay and immunofluorescence staining showed that *Efnb2* mRNA was not colocalized with the satellite cell marker GS (glutamine synthetase) (Figure S1C) or with the marker for microglia‐like tissue‐resident macrophages IBA1 (ionized calcium‐binding adapter molecule) (Figure S1D) in DRGs.

### 2.2 Conditional Ablation of EphB1 Receptors in Vglut2^+^ Neurons Enhances Morphine Antinociception

Studies have shown that EphB1 receptors can regulate excitatory synaptic transmission via interaction with NMDA receptors [23, 24, 25], indicating the vital role of EphB1 receptors in excitatory neurons. Vesicular glutamate transporter‐2 (Vglut2), which mediates the uptake of glutamate into synaptic vesicles in neurons, is the most commonly used molecular marker for excitatory glutamatergic neurons. To further investigate the role of EphB1 receptors in opioid antinociception, we crossed Vglut2‐Cre mice with EphB1^fl/fl^ mice to conditionally knock out EphB1 receptors in Vglut2^+^ excitatory neurons (Figure 2A and Figure S2A). Vglut2 is abundantly expressed in both excitatory interneurons in the DH neurons and DRG neurons [26]. Therefore, we confirmed the knockout efficacy by Western blot analysis that the protein expression of EphB1 receptors in DRGs and the spinal cord was reduced in Vglut2:EphB1^fl/fl^ conditional knockout (cKO) mice (Figure S2B,C). Also, RNA scope assay indicated that the mRNA level of *Ephb1* was reduced in DRG neurons and in Vglut2^+^ DH neurons in Vglut2:EphB1^fl/fl^ cKO mice (Figure S2D,E).

**FIGURE 2:**
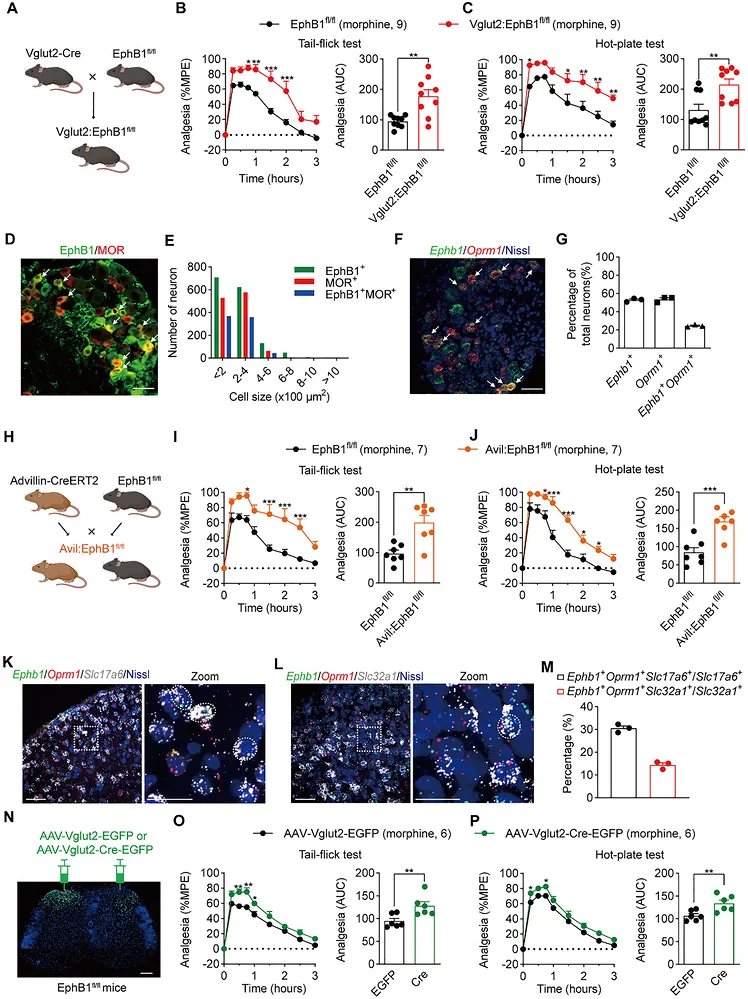
Conditional deletion of EphB1 receptors in DRG neurons or the DH Vglut2^+^ neurons enhances morphine antinociception. (A) Strategy for generating Vglut2:EphB1^fl/fl^ mice. (B, C) Knockout of EphB1 receptors in Vglut2^+^ neurons enhanced the analgesic effect of intrathecal morphine administration (2 nmol) in the tail‐flick test (B) and hot‐plate test (C). Left, %MPE. Right, AUC. Sample sizes were indicated in brackets. (D) Immunofluorescence staining showing coexpression of EphB1 receptors and MORs in DRG neurons. White arrows indicated double‐labeled neurons. Scale bars indicated 50 µm. (E) Area of neurons with EphB1 receptor and MORs expression and co‐expression in DRG neurons. Neurons (1917) from three mice were analyzed. (F, G) RNA scope assay showing colocalization of *Ephb1* mRNA and *Oprm1* mRNA in DRGs. (F) Representative images. White arrows indicated double‐labeled neurons. Scale bars indicated 50 µm. (G) Statistical summary. Neurons (1098) from three mice were analyzed. (H) Strategy for generating Avil:EphB1^fl/fl^ mice. (I, J) Knockout of EphB1 receptors in DRG neurons enhanced the analgesic effect of intrathecal morphine administration (2 nmol) in the tail‐flick test (I) and hot‐plate test (J). Left, %MPE. Right, AUC. Sample sizes were indicated in brackets. (K–M) RNA scope assay showing colocalization of *Ephb1* mRNA and *Oprm1* mRNA in Vglut2^+^ excitatory neurons (*Slc17a6*) (K) or in Vgat^+^ inhibitory neurons (*Slc32a1*) (L) in the DH. (K), (L) Representative images. White circles indicate triple‐labeled neurons. Scale bars indicated 50 µm (left) and 20 µm (right). (M) Statistical summary. Nine spinal cord slices from three mice were analyzed. (N) Schematic illustration of intra‐lumbar spinal dorsal horn injection of AAV‐Vglut2‐EGFP (EGFP) or AAV‐Vglut2‐Cre‐EGFP (Cre) in EphB1^fl/fl^ mice. The scale bar indicated 200 µm. (O, P) Knockout of EphB1 receptors in DH Vglut2^+^ neurons enhanced the analgesic effect of intrathecal morphine administration (2 nmol) in the tail‐flick test (O) and hot‐plate test (P). Left, %MPE. Right, AUC. Sample sizes were indicated in brackets. Statistical analysis by unpaired Student's t‐test or two‐way ANOVA with Bonferroni's multiple comparison test in (B), (C), (I), (J), (O), (P). ^*^ *p *< 0.05, ^**^ *p *< 0.01, and ^***^ *p *< 0.001.

Our behavioral tests showed that conditional deletion of EphB1 receptors in Vglut2^+^ neurons resulted in enhancement of analgesia following morphine administration (2 nmol, i.t.) (Figure 2B,C), while it did not alter baseline nociception (Figure S2F–I). These results indicate that EphB1 receptors in Vglut2^+^ neurons are involved in morphine antinociception. Furthermore, knockout of EphB1 only enhanced the analgesic effect of DAMGO, rather than that of DPDPE and U69593 (Figure S3A–C). These results further suggest a functional interaction between EphB1 receptors and MORs.

Opioids are a mainstay treatment for neuropathic pain and bone cancer pain in clinical treatment. Thus, we further assessed morphine antinociception in the mouse model of pathological pain in EphB1^fl/fl^ and Vglut2:EphB1^fl/fl^ mice. Intrathecal injection of morphine nearly reversed partial sciatic nerve ligation (PSNL)‐induced mechanical allodynia and thermal hyperalgesia in EphB1^fl/fl^ mice, and knockout of EphB1 receptors significantly strengthened morphine antinociception (Figure S3D). Similarly, we established the bone cancer pain (BCP) model by tibial implantation of Lewis lung carcinoma cell line LL/2 (LLC1), and EphB1 receptor knockout also strengthened the analgesic effect of intrathecal morphine administration in BCP mice (Figure S3E).

### 2.3 Deletion of EphB1 Receptors in DRG Neurons and DH Vglut2^+^ Neurons Enhances Morphine Antinociception

As Vglut2 is expressed in both DRG neurons and excitatory interneurons in the spinal cord, we set out to determine whether EphB1 receptors in DRG neurons or in DH Vglut2^+^ neurons are involved in morphine antinociception. We first examine the role of peripheral EphB1 receptors in opioid antinociception. Immunofluorescence staining showed the coexpression of EphB1 receptors and MORs in DRG neurons, and the colocalization was mainly observed in small‐diameter DRG neurons (Figure 2D,E). RNA scope assay also showed a high degree of colocalization of EphB1 mRNA (*Ephb1*) and MORs mRNA (*Oprm1*) in DRG neurons. Quantitative analysis revealed that 52.91% of neurons expressed *Ephb1* mRNAs, 53.72% of neurons expressed *Oprm1* mRNAs, and 24.61% of neurons expressed both mRNAs (Figure 2F,G). Also, immunofluorescence staining showed that co‐expression of EphB1 receptors and MORs was observed on the nerve axons of the dorsal roots and axon terminals in the DH (Figure S4A,B). These results suggest a functional interaction of EphB1 receptors and MORs in DRG neurons and axon terminals.

We continued to determine whether peripheral EphB1 receptors play a role in opioid antinociception, we crossed Advillin‐CreERT2 mice with EphB1^fl/fl^ mice (Avil:EphB1^fl/fl^) to conditionally knock out EphB1 receptors in DRG neurons (Figure 2H and Figure S4C), and Western blot indicated that the protein expression level of EphB1 receptors was reduced in DRGs from Avil:EphB1^fl/fl^ cKO mice (Figure S4D). Also, the RNA scope assay confirmed that the mRNA level of *Ephb1* was reduced in DRG neurons from Avil:EphB1^fl/fl^ mice (Figure S4E). Our behavioral tests showed that deletion of EphB1 receptors in DRG neurons resulted in enhancement of analgesia following morphine administration (2 nmol, i.t.) (Figure 2I,J). Furthermore, ablation of EphB1 receptors in DRG neurons only enhanced the analgesic effect of DAMGO, rather than that of DPDPE and U69593 (Figure S4F–H).

We then further investigated the role of EphB1 in DH neurons in opioid antinociception. RNA scope assay showed that 30.51% Vglut2^+^ excitatory neurons (*Slc17a6* ^+^ neurons) expressed both *Ephb1* mRNA and *Oprm1* mRNA, while only 14.41% of vesicular γ‐aminobutyric acid transporter (Vgat^+^) inhibitory neurons (*Slc32a1* ^+^ neurons) expressed both mRNA in the DH (Figure 2K–M), indicating that *Ephb1* mRNA and *Oprm1* mRNA were mainly colocalized in excitatory neurons, but rarely coexpressed in inhibitory neurons in the DH. These results suggest that EphB1 receptors in excitatory DH neurons mainly contribute to opioid antinociception.

To determine the role of EphB1 receptors in DH Vglut2^+^ neurons in opioid antinociception, we knocked out EphB1 receptors in the lumbar DH Vglut2‐expressing neurons by performing intraspinal injection of a recombinant adeno‐associated virus (rAAV) vector expressing Vglut2‐SV40‐NLS‐Cre‐EGFP in EphB1^fl/fl^ mice, and an rAAV vector expressing Vglut2‐EGFP alone served as a negative control (Figure 2N), and western blot analysis indicated that the protein expression of EphB1 receptors in the spinal cord was significantly reduced following intraspinal injection of rAAV‐Vglut2‐Cre‐EGFP in EphB1^fl/fl^ mice (Figure S5A). Compared with the control AAV‐Vglut2‐EGFP virus‐injected EphB1^fl/fl^ mice, the analgesic effect of intrathecal morphine administration was significantly strengthened in AAV‐Vglut2‐Cre‐EGFP virus‐injected EphB1^fl/fl^ mice (Figure 2O,P). Moreover, the analgesic effect of DAMGO, rather than that of DPDPE and U69593, was enhanced in Vglut2‐Cre‐EGFP virus‐injected EphB1^fl/fl^ mice (Figure S5B–D).

Taken together, we demonstrate that EphB1 receptors in both DRG neurons and the DH Vglut2^+^ neurons modulate MOR‐mediated antinociception, suggesting a functional interaction between EphB1 receptors and MORs.

### 2.4 Conditional Deletion of EphB1 Receptors Enhances MOR Function

We next examined the functional interaction between EphB1 receptors and MORs. Opioids inhibit pain by suppressing the function of voltage‐gated calcium channels (VGCC) in primary sensory neurons [27]. As MORs are predominantly expressed in TRPV1^+^ DRG neurons [21, 22], we recorded the calcium influx induced by VGCC agonist KCl (50 mM) in TRPV1^+^ primary cultured DRG neurons from EphB1^fl/fl^ and Avil:EphB1^fl/fl^ mice. Our results showed that DAMGO‐induced inhibition of calcium influx in TRPV1^+^ neurons was significantly enhanced following deletion of EphB1 receptors in DRG neurons (Figure 3A,B). Moreover, we performed whole‐cell patch clamp to record calcium current in dissociated small‐diameter (< 25 µm) DRG neurons from EphB1^fl/fl^ and Avil:EphB1^fl/fl^ mice. Similarly, DAMGO‐induced suppression of the amplitude of calcium current in DRG neurons was significantly enhanced by ablation of EphB1 receptors in DRG neurons (Figure 3C). These results show that the deletion of EphB1 receptors in DRG neurons strengthened the function of MORs in DRG neurons.

**FIGURE 3:**
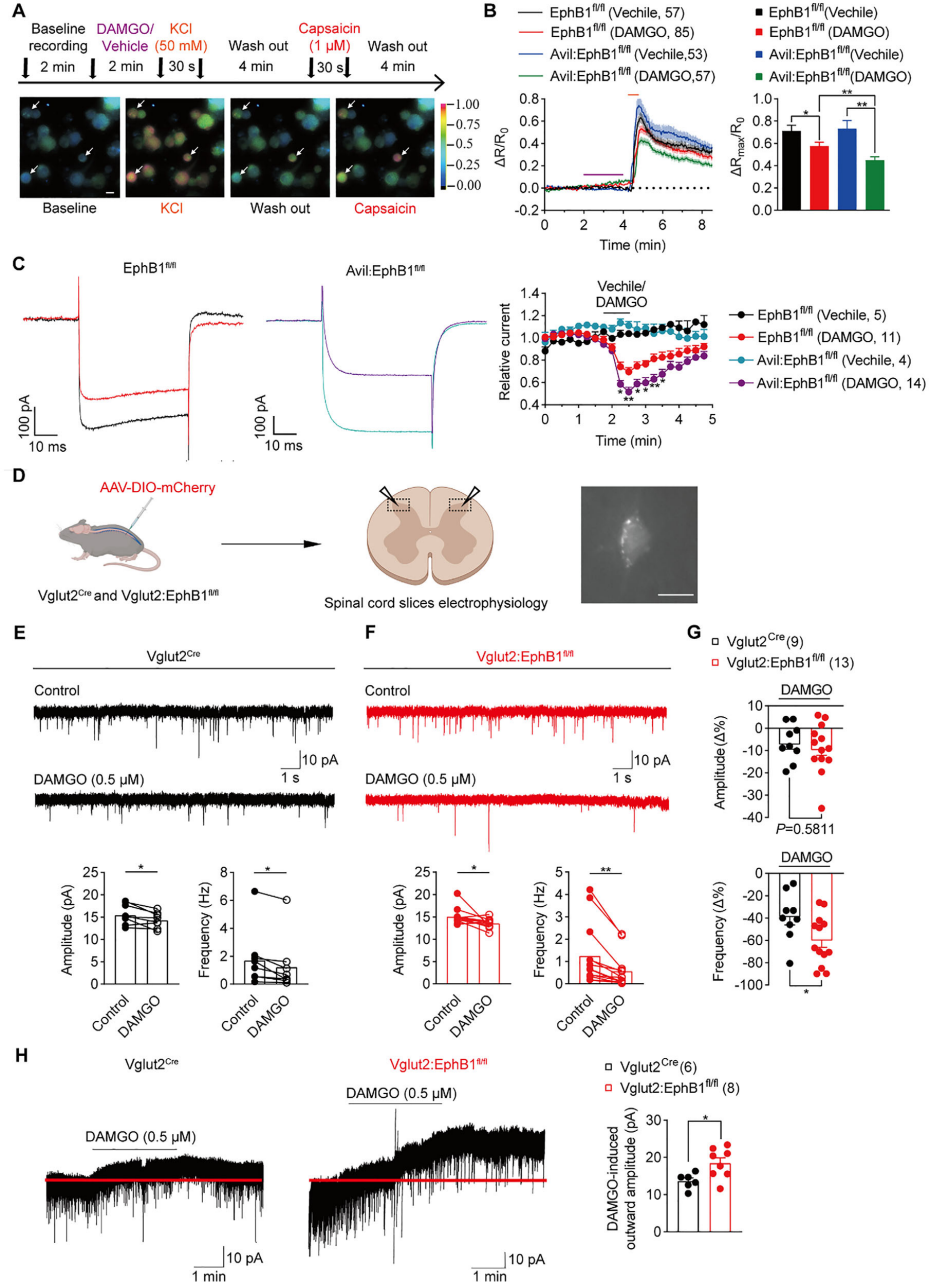
Loss of EphB1 receptors enhances DAMGO‐induced suppression of calcium channels in DRG neurons, inhibition of nociceptive transmission, and activation of GIRK in the DH Vglut2^+^ neurons. (A) Schematic illustration of calcium imaging experiments and representative images of Ca^2+^ responses. KCl (50 mM) was used as the agonist of the VGCC, and capsaicin (1 µM) was used to evoke the TRPV1 channel. White arrows indicated neurons that responded to both KCl and capsaicin. The scale bar indicated 20 µm. (B) Calcium imaging showing that knockout of EphB1 receptors in DRG neurons enhanced the inhibition of calcium influx by DAMGO (0.5 µM) in primary cultured DRG neurons. Left, traces of calcium imaging. Right, quantification of maximum response. The number of neurons used for analysis is indicated in brackets. (C) Whole‐cell patch‐clamp recording showing that knockout of EphB1 receptors in DRG neurons enhanced the suppression of calcium currents by DAMGO (0.5 µM) in primary cultured DRG neurons. Left, traces of calcium currents. Right, time course of relative calcium currents. The number of neurons used for analysis was indicated in brackets. (D) Schematics of intraspinal injection of AAV‐DIO‐mCherry (left) and an example of a Vglut2^+^ DH neuron for spinal cord slices patch‐clamp recordings (right). Scale bar indicates 10 µm. (E, F) Whole‐cell patch‐clamp recordings showing inhibition on sEPSCs by DAMGO (0.5 µM) in Vglut2^+^ DH neurons from EphB1^fl/fl^ (E) and Vglut2:EphB1^fl/fl^ (F) mice. Upper, representative traces. Lower, statistical summary of amplitude (left) and frequency (right). *n* = 9 neurons in Vglut2^Cre^ group, and *n* = 13 neurons in Vglut2:EphB1^fl/fl^ group. (G) Knockout of EphB1 receptors in Vglut2^+^ neurons enhanced DAMGO‐induced inhibition of the sEPSC frequency in DH Vglut2^+^ neurons. Upper, percentage change in the sEPSC amplitude. Lower, percentage change in the sEPSC frequency. The number of neurons used for analysis was indicated in the brackets. (H) Knockout of EphB1 receptors in Vglut2^+^ neurons enhanced DAMGO‐induced outward currents in Vglut2^+^ DH neurons. Left, traces of outward currents. Right, statistical summary. The number of neurons used for analysis was indicated in brackets. Statistical analysis by Paired Student's *t*‐test in (E) and (F), an unpaired Student's *t*‐test in (B), (G), and (H), or a two‐way ANOVA with Bonferroni's multiple comparison test in (C). ^*^ *p *< 0.05 and ^**^ *p *< 0.01.

Inhibition of calcium channels in the central terminals of DRG neurons by opioids also inhibits neurotransmitter release in the DH [5]. To investigate the function of EphB1 receptors on nociceptive synaptic transmission in the DH, we recorded spontaneous excitatory postsynaptic currents (sEPSCs) in outer lamina II (lamina II_o_) neurons in spinal cord slices, which are predominantly excitatory neurons and form a circuit with primary C‐afferents and projection neurons in lamina I [28]. We labeled those neurons by intraspinal injection of AAV‐DIO‐mCherry virus in Vglut2^Cre^ mice or Vglut2:EphB1^fl/fl^ mice (Figure 3D). Our results showed that DAMGO inhibited the frequency and amplitude of sEPSC in Vglut2^+^ DH lamina II_o_ neurons in both EphB1^fl/fl^ and Vglut2:EphB1^fl/fl^ mice (Figure 3E,F). Knockout of EphB1 receptors in Vglut2^+^ neurons significantly enhanced the degree of inhibition on sEPSC frequency, however, no significant difference was observed in the inhibition of sEPSC amplitude in Vglut2:EphB1^fl/fl^ neurons compared to EphB1^fl/fl^ neurons (Figure 3G). These results demonstrate that deletion of EphB1 receptors enhances MOR‐mediated suppression of spinal nociceptive synaptic transmission, indicating promoted MOR function in spinal axon terminals.

Opioids also activate G‐protein coupled inward rectifying potassium channels (GIRK) to generate outward current and hyperpolarization in the DH neurons [29, 30]. We found that DAMGO evoked outward current in Vglut2^+^ DH neurons, and the amplitude of outward current was significantly enhanced by knockout of EphB1 receptors in Vglut2^+^ neurons (Figure 3H). This indicates that EphB1 receptors also modulate the function of MORs expressed in Vglut2^+^ DH neurons.

### 2.5 EphB1 Receptors Control Trafficking and Phosphorylation of MOR at Ser^375^

We then examine the underlying mechanisms by which EphB1 receptors influence MOR function. Given that alterations in opioid receptor trafficking influence opioid antinociception [11], we set out to examine whether EphB1 receptors regulate MOR localization. For this study, we managed to overexpress MOR in Neuro‐2a cells (Figure 4A). Western blot analysis showed that activation of EphB1 receptors by ephrinB2‐Fc (0.01 µg/µL) decreased the membrane expression of MOR and increased its intracellular localization, but did not affect overall MOR expression. Also, ephrinB2‐Fc significantly strengthened DAMGO‐induced internalization of MOR (Figure 4B). Furthermore, immunofluorescence staining showed that MORs were localized on the plasma membrane under basal conditions. EphrinB2‐Fc induced significant internalization of MOR in the absence of MOR agonists, and ephrinB2‐Fc‐induced internalization of MOR was inhibited by the EphB receptor antagonist, STA‐013 (Figure S6A). Also, ephrinB2‐Fc enhanced DAMGO‐induced internalization of MOR (Figure 4C). These results support the idea that EphB1 receptors modulate MOR trafficking.

**FIGURE 4:**
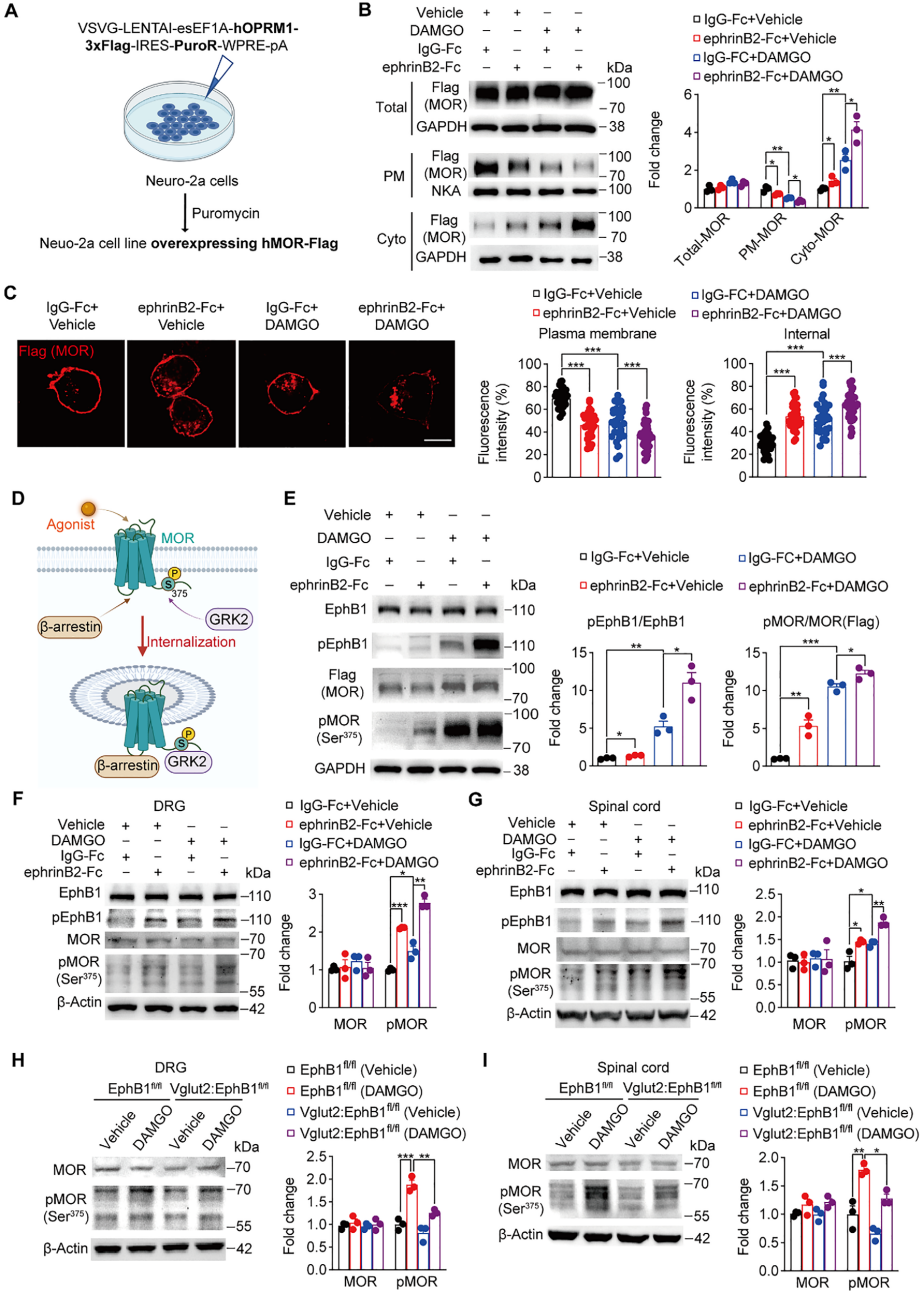
EphB1 receptors regulate MOR trafficking and control the phosphorylation of MOR at Ser^375^. (A) Schematic illustration of generating Neuro‐2a cells overexpressing MOR. (B) Western blot showing the expression of total protein, plasma membrane (PM) distribution, and cytoplasm distribution of MOR in Neuro‐2a cells overexpressing MOR following treatment of ephrinB2‐Fc (0.01 µg/µL). Left, representative bands. Right, statistical summary. Each experiment was repeated three times. (C) Immunofluorescence staining showing MOR internalization in Neuro‐2a cells overexpressing MOR following treatment of ephrinB2‐Fc (0.01 µg/µL). Left, representative images. The scale bar indicates 10 µm. Right, statistical summary. *n* = 36 cells in IgG‐Fc + Vehicle group, *n* = 32 cells in ephrinB2‐Fc + Vehicle group, *n* = 30 cells in IgG‐Fc + DAMGO group, and *n* = 47 cells in ephrinB2‐Fc + DAMGO group. (D) Schematic illustration of GRK2‐mediated phosphorylation of MOR at Ser^375^ and MOR internalization. MOR agonist induces binding of GRK2, which triggers the phosphorylation of MOR at Ser^375^. β‐arrestin is then recruited and mediates the internalization of MOR. (E) Western blot showing increased phosphorylation of EphB1 receptor and MOR at Ser^375^ in Neuro‐2a cells overexpressing MOR following treatment with ephrinB2‐Fc (0.01 µg/µl). Left, representative bands. Right, statistical summary. Each experiment was repeated three times. (F, G) Western blot showing increased phosphorylation of MOR at Ser^375^ in DRGs (F) and spinal cord (G) following intrathecal administration of ephrinB2‐Fc (50 ng). Control IgG‐Fc or ephrinB2‐Fc was intrathecally injected 30 min before intrathecal injection of DAMGO or Vehicle. Left, representative bands. Right, statistical summary. *n* = 3 in each group. (H, I) Western blot showing that knockout of EphB1 receptors in Vglut2^+^ neurons inhibited DAMGO‐induced phosphorylation of MOR at Ser^375^ in DRGs (H) and the spinal cord (I). Left, representative bands. Right, statistical summary. *n* = 3 in each group. Statistical analysis by unpaired Student's *t*‐test in (B), (C), (E), (F), and (G), or one‐way ANOVA with Bonferroni's multiple comparison test in (H) and (I). ^*^ *p *< 0.05, ^**^ *p *< 0.01, and ^***^ *p *< 0.001.

We continued to explore how EphB1 influences MOR trafficking. The termination of G‐protein signaling is initiated by GRKs‐mediated phosphorylation and achieved by arrestin‐mediated internalization [31]. Ligand‐dependent phosphorylation of MOR at the Ser^375^ residue by GRKs has been reported to play a crucial role in the internalization of MOR (Figure 4D) [32, 33, 34, 35, 36, 37]. Thus, we wonder whether EphB1 receptors regulate MOR trafficking via influencing the phosphorylation of MOR. In Neuro‐2a cells overexpressing MOR, ephrinB2‐Fc significantly induced the phosphorylation of MOR at Ser^375^ and also enhanced DAMGO‐induced phosphorylation of MOR at Ser^375^ (Figure 4E). In in vivo experiments, we found that intrathecal injection of ephrinB2‐Fc induced phosphorylation of MOR at Ser^375^ and also significantly augmented DAMGO‐induced phosphorylation of MOR in DRGs and the spinal cord (Figures 4F,G). Conversely, conditional knockout of EphB1 receptors in Vglut2^+^ neurons significantly impaired DAMGO‐induced phosphorylation of MOR at Ser^375^ in DRGs and in the spinal cord (Figure 4H,I). Furthermore, deletion of EphB1 receptors in Vglut2^+^ neurons resulted in inhibition of morphine‐induced phosphorylation of MOR at Ser^375^ in DRGs and in the spinal cord in the mouse model of cancer pain induced by tumor cell implantation (TCI) (Figure S7A,B) and neuropathic pain induced by PSNL (Figure S7C,D). Taken together, these results demonstrate that EphB1 receptors influence phosphorylation of MOR at Ser^375^.

### 2.6 EphB1 Receptors Interact With MOR and GRK2 in DRGs and the Spinal Cord

Multiple studies, using pharmacological and genetic inhibition, have demonstrated the critical role of GRK2/3 in MOR phosphorylation at Ser^375^ by high‐efficacy agonists such as DAMGO, or low‐efficacy agonists such as morphine [10]. Therefore, we wondered whether EphB1 receptors regulate the phosphorylation of MOR through modulating GRK2/3 activity. Co‐immunoprecipitation (Co‐IP) experiments demonstrated that an anti‐EphB1 receptor antibody pulled down GRK2, and anti‐GRK2 antibody also pulled down EphB1 receptors in mouse DRGs and spinal cord lysates (Figure 5A,B), indicating the possible interaction between EphB1 receptors and GRK2. Similarly, co‐IP experiments also demonstrated that an anti‐EphB1 receptor antibody pulled down GRK3 in mouse DRGs and the spinal cord. However, the protein level of GRK3 was much lower than that of GRK2 in mouse DRGs and the spinal cord, as GRK3 was hardly detected by Western blot analysis in DRGs and the spinal cord lysates (Figure S8A,B). Therefore, subsequent analysis was focused on GRK2. In Neuro‐2a cells overexpressing MOR, we confirmed the interaction between EphB1 receptors and GRK2 by co‐IP experiments, and also demonstrated that EphB1 receptors were associated with MORs (Figure 5C). These results suggest that EphB1 receptors, GRK2, and MORs form a protein complex.

**FIGURE 5:**
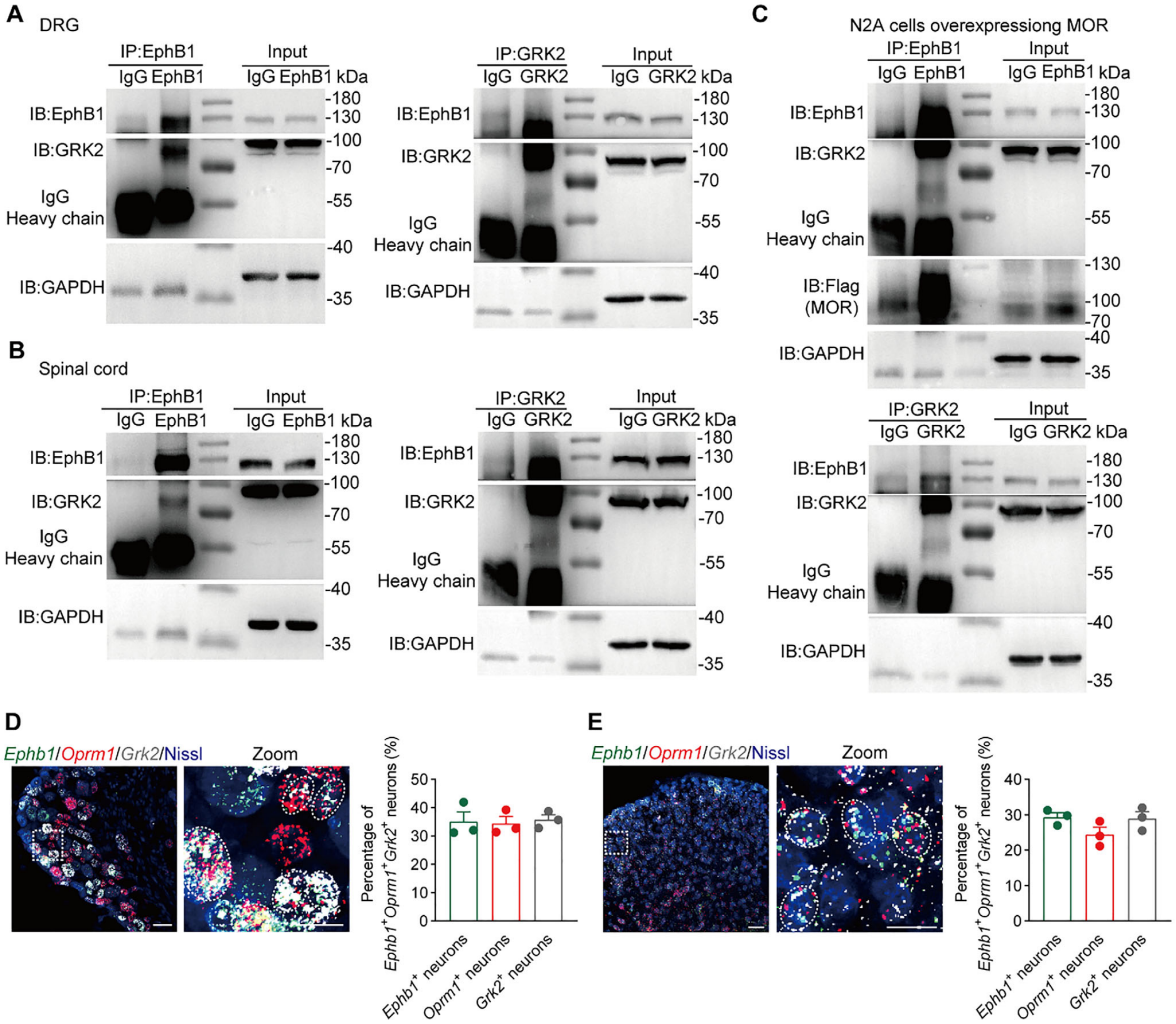
EphB1 receptors interact with MOR and GRK2. (A, B) Co‐IP analysis indicating the interaction between EphB1 receptors and GRK2 in DRGs (A) and the spinal cord (B) in native mice. DRG or spinal cord lysate was immunoprecipitated with control IgG, EphB1 receptor antibody (left), or GRK2 antibody (right), and immunoblotted with EphB1 receptor, GRK2, and GAPDH antibodies. DRG or spinal cord samples were collected from three mice. (C) Co‐IP analysis showing the interaction between EphB1 receptors, GRK2, and MOR in Neuro‐2a cells overexpressing MOR. The cell lysate was immunoprecipitated with control IgG, EphB1 receptor antibody (upper), or GRK2 antibody (lower), and immunoblotted with EphB1 receptor, GRK2, Flag, and GAPDH antibodies. (D, E) RNA scope assay showing colocalization of *Ephb1*, *Oprm1*, and *Grk2* mRNA in DRG neurons (D) or in the spinal cord (E). Left, representative images. White circles indicated triple‐labeled neurons. Scale bars indicate 50 µm (left) and 20 µm (right). Right, statistical summary. DRG neurons (3623) from three mice were analyzed in D, and nine spinal cord slices from three mice were analyzed in E.

Furthermore, we identify the expression pattern of GRK2 in DRGs and the spinal cord. RNA scope and immunofluorescence staining showed that 18.27% and 60.52% of *Grk2* ^+^ neurons co‐expressed CGRP (calcitonin gene‐related peptide) and IB4 (isolectin B4), respectively, whereas only 4.51% of *Grk2* ^+^ neurons were labeled by NF200 (neurofilament‐200), indicating that GRK2 is mainly expressed in DRG nociceptors (Figure S9A). Also, quantitative analysis showed that 34.61% and 49.63% of *Grk2* ^+^ neurons co‐expressed *Slc17a6* or *Slc32a1* in the DH (Figure S9B), demonstrating that GRK2 is expressed in both excitatory and inhibitory DH neurons. These results indicate that the expression pattern of GRK2 in the DRG and spinal cord exhibits cell‐type specificity similar to that of EphB1 receptors and MORs. Furthermore, the RNAscope assay showed that *Ephb1*, *Grk2*, and *Oprm1* mRNA are co‐expressed in DRG neurons (Figure 5D) and in the DH (Figure 5E), further supporting their interaction in DRGs and the spinal cord.

### 2.7 EphB1 Receptors Modulate Internalization and Phosphorylation of MOR at Ser^375^ via GRK2

We further investigate the mechanism of how EphB1 receptors functionally interact with GRK2. Studies have shown that tyrosyl‐phosphorylation of GRK2 modulates its catalytic activity [38, 39, 40]. We wondered whether the EphB1 receptor, a member of RTKs, could phosphorylate GRK2 at tyrosine residues and thus regulate its catalytic activity. The phosphorylation at GRK2 tyrosine residues was assessed by a tyrosine‐phospho‐specific antibody (PY99) after immunoprecipitation with GRK2 antibodies. We found that GRK2 was significantly phosphorylated at tyrosine residues after ephrinB2‐Fc treatment. Meanwhile, ephrinB2‐Fc treatment also enhanced the interaction between GRK2 and MOR (Figure 6A).

**FIGURE 6:**
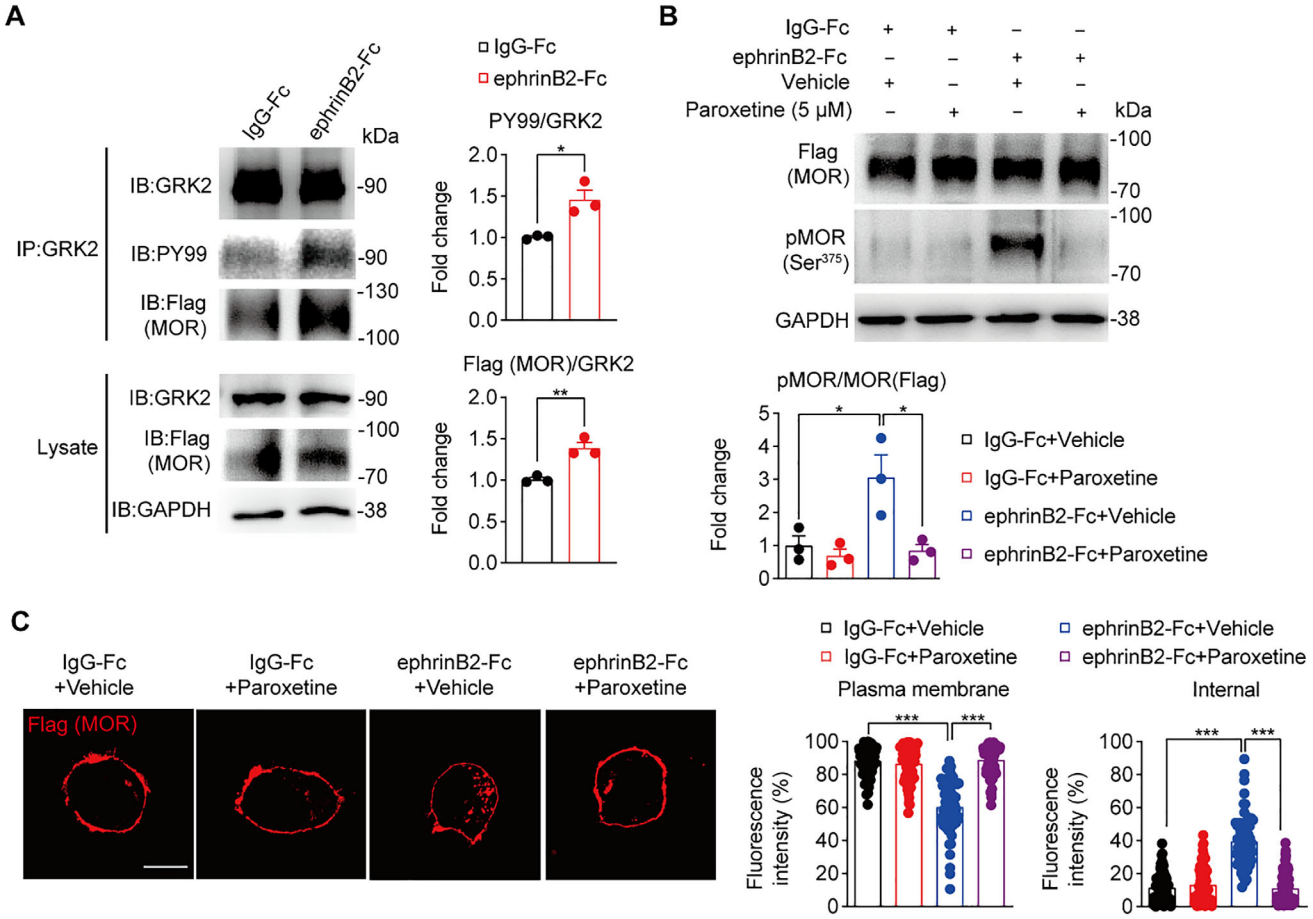
Activation of EphB1 receptors induces phosphorylation of MOR at Ser^375^ and MOR internalization via GRK2. (A) Co‐IP analysis showing increased phosphorylation of GRK2 at tyrosine residues and enhanced interaction between GRK2 and MOR following treatment of ephrinB2‐Fc (0.01 µg/µL) in Neuro‐2a cells overexpressing MOR. The cell lysates were immunoprecipitated with an antibody against GRK2 and immunoblotted with PY99 antibodies to analyze the tyrosine phosphorylation of GRK2, or immunoblotted with Flag antibodies to analyze the interaction between GRK2 and MOR. Left, representative bands. Right, statistical summary. Each experiment is repeated three times. (B) Western blot showing that ephrinB2‐Fc‐induced phosphorylation of MOR at Ser^375^ is inhibited by treatment of the GRK2 inhibitor, Paroxetine (5 µM). Upper, representative bands. Lower, statistical summary. Each experiment is repeated three times. (C) Immunofluorescence staining showing that Paroxetine (5 µM) inhibited ephrinB2‐Fc‐induced internalization of MOR in Neuro‐2a cells overexpressing MOR. Left, representative images. Scale bar indicates 10 µm. Right, statistical summary. *n* = 77 cells in IgG‐Fc + Vehicle group, *n* = 77 cells in IgG‐Fc + Paroxetine group, *n* = 65 cells in ephrinB2‐Fc + Vehicle group, and *n* = 90 in ephrinB2‐Fc + Paroxetine group. Statistical analysis by unpaired Student's *t*‐test in (A) or one‐way ANOVA with Bonferroni's multiple comparison test in (B) and (C). ^*^ *p *< 0.05, ^**^ *p *< 0.01, and ^***^ *p *< 0.001.

A Food and Drug Administration (FDA)‐approved selective serotonin‐reuptake inhibitor, paroxetine, has been identified as a potent and selective GRK2 inhibitor [41]. A previous study showed that paroxetine inhibited DAMGO‐induced internalization of MOR by reducing the phosphorylation of MOR at Ser^375^ [32]. Western blot analysis showed that treatment with paroxetine (5 µM) totally reversed ephrinB2‐Fc‐induced phosphorylation of MOR at Ser^375^ (Figure 6B), and the immunofluorescence staining showed that ephrinB2‐Fc‐induced internalization of MOR was also reversed by paroxetine treatment in Neuro‐2a cells overexpressing MOR (Figure 6C). These results further demonstrate that EphB1 regulates MOR internalization via modulating GRK2‐mediated phosphorylation of MOR at Ser^375^.

## 3 Discussion

This study reveals that EphB1 receptors in DRGs and the spinal cord may drive the development of opioid tolerance by regulating MOR. Morphine administration activates EphB1 receptors in DRGs and the spinal cord. Deletion of EphB1 receptors in DRG neurons or in Vglut2‐expressing DH neurons augments morphine antinociception. Activated EphB1 receptors signaling elicits GRK2‐mediated phosphorylation of MOR at Ser^375^, and thus induce receptor internalization and reduce opioid antinociception.

RTKs constitute a large family of transmembrane receptors activated by selective soluble polypeptide growth factors, cytokines, and hormones. Eph receptors and their ligands, ephrin, represent the largest family of RTKs. EphB receptors orchestrate downstream signaling via tyrosine phosphorylation of target proteins, including Src family kinase (SFK), Ras and RhoA et al. [12, 42], thereby regulating effector proteins. We previously showed that chronic morphine exposure activates EphB1 receptor signaling, and that either pharmacological inhibition or genetic ablation of EphB1 receptors rescues morphine analgesia during tolerance development [17]. Here, we show that acute morphine administration elevates ephrinB2 (an EphB1 ligand) protein levels in DRGs, potentially enabling its binding to and activation of EphB1 receptors in DRGs and the spinal cord. Moreover, selective ablation of EphB1 in dorsal horn Vglut2^+^ excitatory neurons or in DRG neurons potentiated morphine antinociception, highlighting the indispensable role of EphB1 receptors in opioid analgesia. Morphine stimulation upregulated ephrinB2 protein expression in DRGs. However, we were unable to detect this change in the spinal cord, which harbors the central terminals of DRG neurons, by Western blotting—likely owing to limited assay sensitivity and the substantial size of the spinal cord tissue. Our data also showed that morphine increased EphB1 phosphorylation, but did not affect total protein levels, in DRGs, whereas both EphB1 expression and phosphorylation were elevated in the spinal cord. These results point to distinct mechanisms of EphB1 receptor signaling activation in the DRG versus the DH.

Morphine exerts its analgesic effects primarily through MORs, and alterations in MOR signaling profoundly influence antinociceptive outcomes [5]. It is well established that RTKs are closely associated with MOR signaling, thereby regulating analgesic tolerance, physical dependence, and reward [43]. For instance, activation of the insulin receptor (IR) has been shown to potentiate DAMGO‐activated GIRK inward currents via MAPK signaling, possibly through dephosphorylation of MOR tyrosine residues [44]. Additionally, epidermal growth factor (EGF)‐induced activation of EGFR (EGF receptor) promotes MOR phosphorylation by recruiting GRK2 and inducing its translocation to the plasma membrane, ultimately leading to MOR internalization [45]. Conversely, acute treatment with MOR agonists has been shown to transactivate several RTKs, including EGFR [46, 47] and platelet‐derived growth factor receptor (PDGFR) family β [48]. In this study, we demonstrated that EphB1 receptor—a member of the largest family of RTKs—may regulate opioid antinociception through crosstalk with MOR signaling. We demonstrate that acute morphine treatment upregulated ephrinB2 expression in DRGs, via MORs in TRPV1^+^ DRG neurons, and induced EphB1 receptor signaling activation. Conversely, ablation of EphB1 receptors in DRG neurons or in the DH Vglut2^+^ excitatory neurons enhanced MOR function. These data further validate the interaction between MOR signaling and RTKs, suggesting that inhibiting RTKs can be a potential strategy for improving opioid safety.

It is well established that prolonged morphine or other MOR agonist treatment triggers GRK2/3‐mediated phosphorylation of MOR at Ser^375^ [33, 35, 36, 49], leading to β‐arrestin recruitment, receptor internalization, downregulation, and ultimately behavioral tolerance [31]. However, the regulatory mechanisms governing these processes are not yet fully understood. In this study, we report that EphB1 receptor activation led to significant internalization of MOR and phosphorylation of MOR at Ser^375^ independent of MOR agonists, and enhanced MOR agonist‐triggered phosphorylation of MOR and its subsequent internalization. Our RNA scope assay revealed the colocalization of GRK2, EphB1 receptors, and MORs in DRG and the DH neurons. Furthermore, our co‐immunoprecipitation experiments showed a protein interaction among these three molecules and identified GRK2 as the key mediator of EphB1 receptor‐mediated MOR phosphorylation at Ser^375^. Our findings unveil a new mechanism that regulates MOR signaling.

Notably, both GRK2 and GRK3 mediate MOR phosphorylation at Ser^3^ ^7^ ^5^ and internalization in response to the high‑affinity MOR agonist DAMGO, however, studies in HEK293 cells have indicated that GRK2 plays a more predominant role than GRK3 in driving agonist‑induced MOR internalization and β‑arrestin2 recruitment [50]. In this study, our data showed that both GRK2 and GRK3 are capable of interacting with EphB1 receptors, however, the expression of GRK3 protein is significantly lower than that of GRK2 in mouse DRGs and the spinal cord—This leads us to speculate that EphB1 receptors modulate MOR phosphorylation at Ser^3^ ^7^ ^5^ and internalization primarily through GRK2.

GRK2 kinase activity is tightly regulated by multiple mechanisms, including interactions with activated receptors, adaptor proteins, phospholipids, caveolin, and calmodulin, as well as by phosphorylation [51]. GRK2 Phosphorylation at serine residues by protein kinase C (PKC) and protein kinase A (PKA) modulates its cellular localization and kinase activity [52, 53, 54]. Src phosphorylates GRK2 at multiple tyrosine residues and modulates GRK2 activity. Specifically, tyrosine phosphorylation enhances GRK2 kinase activity [38] and promotes its interaction with the Gα subunit of GPCR, thereby inhibiting Gα‐downstream signaling [40]. Conversely, c‐Src‐dependent phosphorylation of GRK2 at specific tyrosine residues mediates its degradation [39]. Furthermore, tyrosine phosphorylation of GRK2 has been implicated in EGFR‐mediated inhibition of dopamine receptor D3 receptor signaling and opioid receptor signaling [45, 55]. Our data show that EphB1 receptor activation phosphorylates GRK2 at tyrosine residues and improves the interaction of GRK2 and MORs. This implies that EphB1 receptor activation promotes the activity of GRK2 via GRK2 phosphorylation at tyrosine residues, though there is a lack of direct evidence. It is of great importance to further investigate the molecular mechanism of how EphB1 receptors mediate the phosphorylation of GRK2 at tyrosine residues and thereby modulate GRK2 activity. Given that GRK2 also mediates the termination of other signaling of GPCRs, it is plausible that EphB1 signaling, perhaps as well as other members of ephrin‐Eph signaling, may serve as general regulators of GPCRs function and trafficking in other processes. Understanding how ephrin‐Eph signaling regulates the enormous repertoire of over 800 GPCRs now becomes an expansive and exciting area for future investigation.

General mechanisms of GPCR signaling activation by RTKs involve the recruitment of GPCR signaling effectors such as GRKs [56] and β‐arrestins [57, 58]. As described in previous studies, inhibition of Src kinase, a key downstream signaling molecule of EphB1 receptors, attenuates MOR internalization through its interaction with β‐arrestin2 [59, 60]. Thus, we propose that EphB1 receptors might regulate MOR trafficking via other GPCR signaling effectors. Moreover, multiple studies have demonstrated that activation of RTK downstream kinases, including phosphoinositide 3‐kinase (PI3K), protein kinase C (PKC), protein kinase B (Akt/PKB), and Src [38, 61], regulates GPCR phosphorylation and desensitization. Accordingly, we cannot exclude the involvement of EphB1 downstream signaling effectors in EphB1 receptors‐mediated, GRK2‐dependent MOR phosphorylation and internalization. Future investigations into the role of EphB1 receptor downstream kinases in these processes would be of considerable interest.

## 4 Conclusion

Our findings demonstrate that EphB1 receptors modulate opioid antinociception by regulating GRK2‐mediated MOR trafficking. Morphine administration activates EphB1 receptors in DRG and the spinal cord. EphB1 receptors in DRG neurons and Vglut2^+^ DH neurons interact with GRK2 and induce GRK2‐mediated phosphorylation of MOR at Ser^375^ and the subsequent MOR internalization, thereby reducing morphine antinociception. This study provides a novel molecular target for preventing and/or reversing opioid tolerance.

## 5 Methods

### 5.1 Animals, Drugs and Drug Administration

All experimental protocols were approved by the Southern University of Science and Technology Animal Care and Use Committee. C57BL/6J mice were supplied by Guangdong Medical Laboratory Animal Center. EphB1‐flox (S‐CKO‐09887) mice were purchased from Cyagen. Vglut2‐Cre (#028863) mice, Advillin‐CreERT2 (#032536), TRPV1‐Cre (#017769), and Oprm1‐flox (#030074) mice were purchased from The Jackson Laboratory. Mice of both sexes aged 6 to 8 weeks were used for electrophysiological studies on the spinal cord and DRG neurons. Mice of both sexes aged 8 to 10 weeks, including conditional knockout mice and corresponding wild‐type control mice, were used for behavioral and pharmacological studies.

Morphine hydrochloride injection was purchased from Northeast Pharm. DAMGO (HY‐P0210), DPDPE (HY‐P1334), Paroxetine (HY‐122272), and STA‐013 (HY‐179572) were purchased from MedChemExpress; U69593 (U103) was purchased from Sigma; Recombinant mouse ephrinB2‐Fc (496‐EB‐200) was purchased from R&D Systems; control mouse IgG‐Fc (JAC‐015‐000‐008) was purchased from Jackson ImmunoResearch. All of these drugs were reconstituted following the manufacturer's instructions. Working solutions were prepared from stocks on the day of use. The drug dosages were chosen based on published studies and preliminary experiments. In the behavior experiments, morphine (2 nmol), DAMGO (0.1 nmol), U69593 (10 nmol), and DPDPE (20 nmol) were delivered intrathecally in 5 µL by lumbar puncture at the intervertebral space of L5‐L6 during anesthesia with isoflurane, and ephrinB2‐Fc or control IgG‐Fc (50 ng) was delivered intrathecally 30 min before morphine, DAMGO, U69593, or DPDPE administration. In the electrophysiology experiments, DAMGO (0.5 µM) was applied during recording. In the in vitro test, Neuro‐2a cells were treated with DAMGO (0.1 µM), ephrinB2‐Fc (0.01 µg/µL), STA‐013 (50 µM), or paroxetine (5 µM) simultaneously for 30 min.

### 5.2 Intraspinal Injection of Viruses

Mice were fixed in a stereotaxic frame under anesthesia. A pair of microscissors was used to make a 2‐cm incision at the hump and to cut the musculature from the base of the head through the midline of the back, so as to expose the underlying vertebral column. The rostral and caudal vertebral column was attached by custom‐made clamps. Then, the paraspinal muscles around the hump were removed to expose the vertebrae by incising the dura mater and the arachnoid membrane. A glass micropipette filled with viruses was inserted into spinal segments between T11‐12, T12‐13, and T13‐L1 vertebrae (500 µm lateral from the midline and 300 µm in depth from the surface of the dorsal root entry zone) to target neurons in the lumbar spinal cord (L1‐L6 spinal segments). For knocking out EphB1 receptors in the Vglut2^+^ DH neurons, a volume of 300 nL of rAAV‐Vglut2‐SV40 NLS‐Cre‐EGFP and control rAAV‐Vglut2‐EGFP (2 × 10^12^ vg/mL) from Braincase was delivered. For targeting Vglut2^+^ DH neurons, a volume of 300 nL of AAV‐DIO‐mCherry (2 × 10^12^ vg/mL) from Braincase was delivered. The virus was injected at 100 nL/min via a glass micropipette connected to an infusion pump. The micropipette remained in place for 5 min to prevent leakage of virus, and the skin was sutured after virus injection.

### 5.3 Mouse Models of Neuropathic and Bone Cancer Pain

Neuropathic pain was mimicked in a mouse model of PSNL. In the mid‐thigh, the sciatic nerve was exposed, and half of it was lightly ligated with 4‐0 silk, and the wound was closed. Bone cancer pain was mimicked by tumor cell implantation (TCI) into the tibia. LLC1 (Keygen, KG070) cells were suspended in phosphate‐buffered saline (PBS) at 1 × 10^8^/ml before inoculation. Following isoflurane anesthesia, the patellar ligament was exposed after making a superficial 0.5 to 1 cm incision adjacent to the knee joint. Then, a Hamilton microsyringe was inserted into the tibia cavity through the intercondylar notch, and 2 µL of suspension was slowly injected into the tibia cavity. The microsyringe remained in place for 2 min to prevent leakage of tumor cells. Sham surgery mice underwent a similar procedure with injecting boiled cells. Lastly, the wound was closed with silk sutures.

### 5.4 Behavioral Tests

Before baseline measurements, animals were acclimated to the testing environment for 2–3 days. All the tests were conducted blindly. In the tail‐flick test, mice were restrained in a tube with air holes. The tail‐flick latency was measured from immersion of the distal 3 cm of the tail in 50°C hot water until the tail flicked or was removed from hot water, with a 15 s cutoff value to avoid thermal injury. The tail‐flick latency was measured before and after drug injection. Data are expressed as the percent maximum possible effect (MPE) (%) = 100 × [(post‐drug response − baseline response)/(cutoff response − baseline response)]. The MPE (%) data from each animal were then converted to area under the curve (AUC). In the hot‐plate test, the hot‐plate latency was measured from the time that mice were placed on a 55°C hot plate until exhibiting signs of pain avoidance, such as jumping, paw lifting, flinching, or licking. Then, mice were quickly removed from the hot plate. A 40 s cutoff value was applied to avoid thermal injury.Each animal was tested three times, and the average hot‐plate latency was used for data analysis. Data are expressed as the MPE, and the MPE data from each animal were converted to AUC.

The protocols for measuring painful behavior were similar to those described previously [62]. Mechanical sensitivity was assessed by the von Frey test (Aesthesio, Danmic Global). Animals were individually enclosed in boxes on an elevated metal mesh. The mechanical force was applied with a series of von Frey hairs with logarithmically increasing stiffness (0.02 to 2 g), with a 5 min interval between trials. The force was applied perpendicularly to the central plantar surface of the hind paw for approximately 2 s. The paw withdrawal threshold was determined using the up‐down method. For measuring thermal sensitivity, animals were placed individually in transparent plastic boxes on a glass floor held at 30°C. Radiant heat was applied to the plantar surface of the hind paw with a plantar stimulator analgesia meter (Woodland Hills, IITC model 390G), with a 20 s cutoff time to avoid thermal injury. The paw withdrawal latency was recorded when mice responded to heat stimulation. Each animal was tested three times, with a 5 min interval between trials. The average paw withdrawal latency was used for data analysis.

### 5.5 Protein Determination

Protein abundance was determined by Western blot analysis. Deeply anesthetized mice were perfused transcardially with PBS, and lumbar spinal cord and DRG tissues were rapidly collected and stored at −80°C. Tissue samples and Neuro‐2a cells were lysed in ice–cold (0°C) RIPA (Radio immunoprecipitation assay) lysis buffer supplemented with protease inhibitors and phosphatase inhibitors for 30 min. The lysates were then centrifuged at 12 000 rpm for 15 min, and the supernatant was retained. To analyze the membrane fraction in Neuro‐2a cells, the samples were prepared using a Minute Plasma Membrane Protein Isolation and Cell Fractionation Kit (SM‐005, Invent Biotechnologies) according to the manufacturer's instructions. Protein concentration was determined by the bicinchoninic acid (BCA) test, and the total protein content between samples was equalized. Protein samples were separated by SDS‐PAGE (Sodium dodecyl sulfate‐polyacrylamide gel electrophoresis) and transferred to a 0.2 or 0.45 µm polyvinylidene fluoride (PVDF) membrane (Millipore, ISEQ00010 and IPVH00010). The membranes were then incubated overnight at 4°C with primary antibodies against target proteins, including anti‐EphB1 (Abcam, ab129103, 1:1000), anti‐pEphB1(Abcam, ab61791, 1:1000), anti‐ephrinB2(Abcam, ab131536, 1:1000), anti‐Mu Opioid Receptor (Abcam, ab134054, 1:1000), anti‐Phospho‐Mu‐Opioid Receptor (Ser^375^) (Cell Signaling Technology, 3451, 1:1000), anti‐DYKDDDDK (Flag) Tag (Cell Signaling Technology,14793, 1:1000), anti‐GRK2 (Cell Signaling Technology, 74761, 1:1000), anti‐GRK3 (Cell Signaling Technology, 80362, 1:1000), anti‐Na^+^/K^+^‐ATPase α1 (Cell Signaling Technology, 3010, 1:1000), anti‐β‐Actin (Cell Signaling Technology, 4970, 1:1000), anti‐GAPDH (Sangon, D110016, 1:1000), and anti‐PY99 (Santa Cruz Biotechnology, sc‐7020, 1:100), followed by 3 × 10 min washing by Tris‐buffered saline with 0.1% Tween‐20 (TBST) at room temperature. The membrane was then incubated with peroxidase‐labeled secondary antibody (Sangon, D110058, 1:10000) at room temperature for 1 h, and washed for 10 min by TBST three times. The membranes were then developed by enhanced chemiluminescence (ECL) plus, and images were acquired by a chemiluminescence instrument (Tanon). Data were analyzed with the associated software TanonImage.

### 5.6 Co‐Immunoprecipitation

Neuro‐2a cells, lumbar spinal cord segments, and DRG tissues were lysed in ice‐cold NP‐40 (Nonidet P‐40) lysis buffer supplemented with protease inhibitors and phosphatase inhibitors for 60 min. The lysates were centrifuged at 12 000 rpm for 15 min, and the supernatant was collected for Co‐IP. Lysates were immunoprecipitated with 1 µg control rabbit IgG, anti‐EphB1 (Abcam, ab129103), or anti‐GRK2 antibody (Cell Signaling Technology, 74761), and the mixtures were rotated overnight at 4°C. Then, the lysates were incubated with Protein A/G Magnetic Beads (MedChemExpress, HY‐K0202), which were rinsed five times by PBS with 0.1% Tween‐20 (PBST) before use, and were rotated at 4°C for 6 h. The complexes were washed six times with PBST and denatured by adding 1x loading buffer and incubation at 95°C for 5 min. Western blot analysis was used to detect the IP complexes further.

### 5.7 Immunohistochemistry

Deeply anesthetized mice were transcardially perfused with PBS followed by 4% paraformaldehyde (PFA). Lumbar spinal cord and DRGs were collected and postfixed in 4% PFA overnight at 4°C, followed by dehydration in 30% sucrose for three days at 4°C. Tissues were embedded in optimal cutting temperature (OCT) compound and stored at ‐80°C. Tissues were thawed at −20°C for at least 1 h before slicing, and spinal slices (20 µm) and DRG slices (15 µm) were prepared with a microtome cryostat (Leica, CM1520). Sections were blocked for 2 h in PBS containing 10% donkey serum, and 0.3% Triton X‐100, and were then incubated overnight at 4°C with primary antibodies, including anti‐EphB1 (Santa Cruz Biotechnology, sc‐130054, 1:50), anti‐Mu opioid receptor (Abcam, ab134054, 1:100), anti‐GS (Sigma, MAB302, 1:400), anti‐IBA1 (Wako, 016–20001, 1:400), anti‐CGRP (Abcam, ab81887, 1:400), anti‐IB4 (Sigma, L2895, 1:50), and anti‐NF200 (Sigma, N0142, 1:400). After 3×5 min by PBS, sections were incubated by secondary antibody (Jackson ImmunoResearch, donkey anti‐mouse IgG conjugated with Alexa Fluor 488 or Cy3, and donkey anti‐rabbit IgG conjugated with Alexa Fluor 488 or Cy3, 1:500) or Nissl staining (Invitrogen, N21479, 1:200) for 2 h at room temperature if needed. Then, the slices were rinsed for 5 min by PBS three times. The slices were incubated with 4'6'‐diamidino‐2‐phenylindole (DAPI) (Sigma, D9542, 1:10000) for 5 min at room temperature if needed. Lastly, sections were mounted with anti‐fade mounting medium (BBI Life Sciences, E67501‐0010) before observation by using a confocal microscope (Zeiss, LSM980). For analyzing MOR internalization, the fluorescent intensity of MOR in the whole cell and in the cytoplasm was determined by ImageJ, and the fluorescent intensity of MOR on the cell membrane was calculated by subtracting the fluorescent intensity of MOR in the cytoplasm from that of the whole cell.

### 5.8 RNAscope In Situ Hybridization

RNA scope staining was performed with RNAscope Multiplex Fluorescent Detection Assay V2 (Advanced Cell Diagnostics, 323100) according to the manufacturer's protocol. Spinal cord and DRG sections were dehydrated by ethanol and incubated with hydrogen peroxide at room temperature for 10 min, followed by treatment with Protease III at 40°C for 30 min. Then, commercial probes against *Efnb2* (477671), *Ephb1* (567571‐C3), *Oprm1* (315841‐C2), *Grk2* (551601), *Slc17a6* (319171, 319171‐C2), and *Slc32a1* (319191, 319191‐C2) from Advanced Cell Diagnostics and signal amplifier were applied. Fluorescence signals were generated by using the Fluorescein Evaluation Kit from PerkinElmer (NEL760001KT) according to the manufacturer's instructions, followed by immunofluorescence staining if co‐detection of protein markers was needed. Images were acquired by using a confocal microscope (Zeiss, LSM980). For immunohistochemistry and RNA scope, lumbar spinal cord tissues were evenly cut into three segments before embedding. Three spinal cord slices from one mouse were obtained simultaneously and used for immunohistochemistry and RNA scope. Three images of spinal cord sections per mouse were analyzed, and the average data were used to represent data from one mouse. Similarly, L1‐L6 lumbar DRGs were sliced and used for the experiment, and the average data from one mouse were used to present. The average data obtained from three mice were shown and analyzed.

### 5.9 Cell Culture and Transfection

LLC1 were obtained from Keygen, and Neuro‐2a cells were purchased from TransGen Biotech. Cells were cultured in Dulbecco's modified Eagle medium (DMEM) (21068028, Gibco, Thermo Fisher Scientific), containing 10% fetal bovine serum (FBS) (10099141C, Gibco, Thermo Fisher Scientific) and 1% Penicillin‐Streptomycin (15140122, Thermo Fisher Scientific). Neuro‐2a cells were planted in six‐well plates at 1 × 10^5^ per well on the day before transfection. Cells were then transfected with VSVG‐LENTAI‐esEF1A‐hOPRM1‐3xFlag‐IRES‐PuroR‐WPRE‐pA (Taitool Bioscience) for hMOR overexpression, while using polybrene (6 µg/µL) to help with virus transfection, and were incubated for 24 h at 37°C. Then, an equal volume of complete culture medium was added to reduce the concentration of polybrene. After culturing for another 24 h at 37°C, cells were cultured in the complete medium containing 3 µg/µL puromycin (Beyotime, ST551) for one week to establish a stable Neuro‐2a cell line overexpressing hMOR.

### 5.10 Primary Culture of DRG Neurons

The protocol for primary DRG neuron culture was similar to that we previously reported [19]. DRGs from young mice (6∼8 weeks) were quickly collected and digested for 60 min at 37°C by 1.25 mg/mL collagenase type IV (Sigma, C5138) and 2.5 mg/mL dispase II (Sigma, D4693), which were dissolved in Hank's balanced salt solution (HBSS). Tissues were then mechanically dissociated by pipetting, and the complexes were filtered through a 70‐µm cell strainer. The cell suspension was centrifuged at 1000 rpm for 5 min. The supernatant was discarded, and the pellet was resuspended in Neurobasal medium (Thermo Fisher Scientific, Gibco, 10888‐022) containing 10% FBS, 2% B‐27 supplement (Thermo Fisher Scientific, Gibco, 17504044), and 1% Penicillin‐Streptomycin. Then, isolated cells were plated on coverslips, which were pre‐coated with 0.1 mg/mL poly‐D‐lysine (Millipore, A‐003‐E) overnight, and grown in complete culture medium at 37°C for at least 24 h before use.

### 5.11 Calcium Imaging

The primary cultured DRG neurons were loaded with 5 µM Fura‐2AM (Beyotime, S1052) diluted in Ringer's solution that consists of (in mM)130 NaCl, 2.5 CaCl_2_, 3 KCl, 0.6 MgCl_2_, 10 HEPES (N‐(2‐Hydroxyethyl) piperazine‐N'‐2‐ethanesulfonic acid), and 10 Glucose (pH = 7.4 adjusted with NaOH) for 45 min. During recording, the coverslips containing DRG neurons were placed in the recording chamber and continuously perfused with Ringer's solution. Digitized images of fluorescence at 340 nm and 380 nm excitation in the DRG neurons, but not the glial cells, were captured by a fluorescence microscope (Hamamatsu) every 2 s, and the intensity was measured by Metafluor software (Molecular Device). The fold change of intracellular concentration of Ca^2+^ ([Ca^2+^]_i_) was determined by the ratio of 340/380 nm emission fluorescence intensity. Data were presented as ΔR/R_0_, determined as the fraction ΔR (R_t_‐R_0_) of the increase in a given ratio over the baseline ratio (R_0_). The baseline ratio (R_0_) was defined as the average 340/380 ratio before drug application. Those cells with a ΔR/R_0_ > 0.05 were defined as responsive cells.

### 5.12 Whole‐Cell Patch‐Clamp Recording in Primary Cultured DRG Neurons

During recording, the coverslips containing DRG neurons were placed in the recording chamber and continuously perfused with external solution containing (in mM) 135 Tetraethylammonium (TEA)‐Cl, 1 CaCl_2_, 10 HEPES, 4 MgCl_2_, and 0.1 µM tetrodotoxin (TTX) (pH 7.4 adjusted with TEA‐OH, 300 mOsm). The patch pipettes were pulled from borosilicate capillaries (Sutter Instruments) by a micropipette puller (Sutter Instruments, P1000) and filled with pipette solution for calcium current recording containing (in mM) 126 CsCl, 5 Mg‐adenosine triphosphate (ATP), 10 ethylene glycol tetraacetic acid (EGTA), and 10 HEPES (pH = 7.3 adjusted with CsOH, 300 mOsm). Pipette resistance was 6–8 MΩ. The calcium current was evoked by a 40‐ms step depolarization from −80 to −10 mV and recorded by an EPC‐10 amplifier and Patchmaster software (HEKA Elektron). The data were stored and analyzed by Clampfit 10.6. The relative current is defined as the ratio of the amplitude of calcium current at each time point to the average of baseline amplitude.

### 5.13 Whole‐Cell Patch‐Clamp Recording in Mouse Spinal Cord Slices

In deeply anesthetized mice of both sexes (aged 6∼8 weeks), a micro‐scissors was used to remove the skin of the back and cut the vertebral column to expose the spinal cord. The lumbar spinal cord was quickly removed, and the spinal pia mater was carefully removed with tweezers. The spinal cord was fixed in an agar gel, and transverse spinal slices (350 µm) were prepared with a vibrating microslicer (Leica, VT1200S) in ice‐cold dissection solution consisting of (in mM) 25 NaHCO_3_, 2.5 KCl, 1.25 NaH_2_PO_4_, 0.5 CaCl_2_, 3.5 MgCl_2_, and 240 Sucrose (equilibrated with 95% O_2_ and 5% CO_2_). The spinal cord slices were then incubated at 32°C for at least 30 min for recovery in artificial cerebrospinal fluid (ACSF) containing (in mM) 126 NaCl, 3 KCl, 1.3 MgCl_2_, 2.5 CaCl_2_, 26 NaHCO_3_, 1.25 NaH_2_PO_4_, and 11 Glucose (equilibrated with 95% O_2_ and 5% CO_2_). During recording, the spinal cord slices were placed in the recording chamber and continuously perfused with oxygenated ACSF at room temperature. Lamina II neurons were located in a translucent band in the spinal cord dorsal horn under a microscope (Hamamatsu) with light transmitted from below. The pipette solution for recording sEPSCs and opioid‐induced outward current contained (in mM)135 K‐gluconate, 5 KCl, 0.5 CaCl_2_, 2 MgCl_2_, 5 EGTA, 5 HEPES, and 5 Mg–ATP (pH 7.3 adjusted with KOH, 300 mOsm). The pipettes had a resistance of 8 to 10 MΩ. The sEPSC and opioid‐induced outward currents were recorded by voltage clamp at a holding potential of −70 mV. sEPSC events were analyzed using Mini Analysis Program version 6.0.3. Opioid‐induced outward currents were analyzed by Clampfit 10.6. The outward current value was defined as the difference between the peak amplitude value of an outward current and the amplitude of the baseline current under voltage clamp.

### 5.14 Statistics

GraphPad Prism version 9.5 was used to perform all statistical analyses. Statistical significance was defined as follows: not significant (n.s.) for *p* > 0.05; ^*^ *p *< 0.05; ^**^ *p *< 0.01; and ^***^ *p *< 0.001. For comparing two groups, the Student's *t* test was employed. For comparisons involving more than two groups, one‐way analysis of variance (ANOVA) was used, whereas two‐way ANOVA was used for experiments with two independent variables. All data are presented as mean ± standard error of the mean (SEM).

## Author Contributions

**Bo Peng**: writing – review and editing, writing – original draft, project administration, conceptualization, methodology. **Xiaqing Lin**: methodology, formal analysis. **Hongyu Yao**: formal analysis, methodology. **Cheng Li**: formal analysis, software, data curation. **Huabao Liao**: software, formal analysis, data curation. **Yuexin Liu**: software, formal analysis, data curation. **Boxu Cao**: software, data curation, formal analysis. **Xue‑Jun Song**: writing – review and editing, writing – original draft, funding acquisition, project administration, supervision, conceptualization, validation.

## Conflicts of Interest

The authors declare no conflicts of interest.

## Supporting information




**Supporting File**: advs78099‐sup‐0001‐SuppMat.docx.

## Data Availability

All data related are included in the manuscript and will be available from the corresponding author on reasonable request.
