# Supplementary material for: EphB1 Receptor Blockade Augments Morphine Antinociception via Inhibiting GRK2‐Mediated µ‐Opioid Receptor Internalization
Source: Adv Sci (Weinh). 2026 Sep 30:e78099. Online ahead of print. doi: 10.1002/advs.78099 (PMC13627775; doi:10.1002/advs.78099)
Supplement: Supplementary file 1 [file ADVS-9999-e78099-s001.docx]

Supporting Information

EphB1 receptor blockade augments morphine antinociception via inhibiting GRK2-mediated μ-opioid receptor internalization

*Bo Peng, Xiaqing Lin, Hongyu Yao, Cheng Li, Huabao Liao, Yuexin Liu, Boxu Cao, and Xue‑Jun Song**

**Figure S1.** Conditional deletion of MOR in TRPV1^+^ DRG neurons inhibits morphine-induced increase in ephrinB2 protein level in DRGs. **A** Western blot showing that morphine-induced increase in ephrinB2 in DRGs was reversed by conditional ablation of MOR in TRPV1^+^ DRG neurons. Left, representative bands. Right, statistical summary. n = 3 in each group. **B**-**D** RNA scope assay and immunofluorescence staining showing colocalization of *Efnb2* mRNA with *Oprm1* mRNA (**B**), GS (**C**), or IBA1 (**D**) in DRGs. Left, representative images. White arrows indicated double-labeled neurons. Scale bars indicated 50 μm. Right, statistical summary. Neurons (4224) from three mice were analyzed in **B**. Statistical analysis by unpaired Student *t* test and one-way ANOVA with Bonferroni’s multiple comparison test in (A). ***P* < 0.01 and ****P* < 0.001.

**Figure S2.** Conditional deletion of EphB1 receptors does not change the basal pain. **A** Representative images of the genotyping result of EphB1^fl/fl^ and Vglut2:EphB1^fl/fl^ mice. **B**, **C** Western blot showing that the protein expression of EphB1 receptors in DRGs (**B**) and the spinal cord (**C**) was inhibited by EphB1 receptor knockout in Vglut2^+^ neurons. Left, representative bands. Right, statistical summary. n = 3 in each group. **D**, **E** RNA scope assay showing that the mRNA level of *Ephb1* in DRG neurons (**D**) and in DH Vglut2^+^ neurons (**E**) was inhibited by EphB1 receptor knockout in Vglut2^+^ neurons. Left, representative images. Right, statistical summary. (**D**) Scale bars indicated 50 μm. Neurons (597) from three mice in EphB1^fl/fl^ group, and neurons (450) from three mice in Vglut2:EphB1^fl/fl^ group were analyzed. (**E**) Scale bars indicated 10 μm. White circles indicated *Ephb1* mRNA-expressing Vglut2^+^ neurons. Nine slices from three mice in each group were analyzed. **F**-**I** Conditional deletion of EphB1 receptors did not alter basal pain threshold in von Frey test (**F**), Hargreaves test (**G**), hot-plate test (**H**), and tail-flick test (**I**). Sample sizes were indicated in brackets. Statistical analysis by unpaired Student *t* test in (B) to (I). **P* < 0.05, ***P* < 0.01, and ****P* < 0.001.

**Figure S3.** Conditional ablation of EphB1 receptors in Vglut2^+^ neurons enhances MOR-mediated antinociception and morphine antinociception in mouse model of pathological pain. **A**-**C** Knockout of EphB1 receptors in Vglut2^+^ neurons enhanced the analgesic effects of intrathecal administration of DAMGO (**A**), but not that of DPDPE (**B**) or U69593 (**C**), in the tail-flick test (left) and hot-plate test (right). Upper, %MPE. Lower, AUC. **D**, **E** Knockout of EphB1 receptors in Vglut2^+^ neurons enhanced the analgesic effects of intrathecal morphine administration (2 nmol) in mice with PSNL-induced neuropathic pain (**D**) and TCI-induced bone cancer pain (**E**). Left, schematic illustration for inducing PSNL-induced neuropathic pain and TCI-induced BCP in mice. Right, measurement of mechanical and thermal sensitivity. **A**-**E** Sample sizes were indicated in brackets. Statistical analysis by unpaired Student *t* test and two-way ANOVA with Bonferroni’s multiple comparison test in (A) to (E). **P* < 0.05, ***P* < 0.01, and ****P* < 0.001. ^#^*P* < 0.05 and ^##^*P* < 0.01.

**Figure S4.** Deletion of EphB1 receptors in DRG neurons enhances MOR-mediated antinociception. **A**, **B** Immunofluorescence staining showing colocalization of EphB1 receptors and MORs in dorsal roots (**A**) and central terminals in the DH (**B**). White arrows indicated double-labeled dorsal roots. Scale bar indicated 50 μm. **C** Representative images of the genotyping result of EphB1^fl/fl^ and Avil:EphB1^fl/fl^ mice. **D** Western blot showing that the protein expression of EphB1 receptors in DRGs was inhibited by DRG neuron-specific EphB1 receptor knockout. Left, representative bands. Right, statistical summary. n = 3 in each group. **E** RNA scope assay showing that the mRNA level of *Ephb1* in DRG neurons was inhibited by DRG neuron-specific EphB1 receptor knockout. Left, representative images. Scale bars indicated 50 μm. Right, statistical summary. Neurons (429) from three mice in EphB1^fl/fl^ group and neurons (421) from three mice in Avil:EphB1^fl/fl^ group were analyzed. **F**-**H** Knockout of EphB1 receptors in DRG neurons enhanced the analgesic effects of intrathecal administration of DAMGO (**F**), but not that of DPDPE (**G**) or U69593 (**H**), in the tail-flick test (left) and hot-plate test (right). Upper, %MPE. Lower, AUC. Sample sizes were indicated in brackets. Statistical analysis by unpaired Student *t* test in (D) to (H), or two-way ANOVA with Bonferroni’s multiple comparison test in (F) to (H). **P* < 0.05, ***P* < 0.01, and ****P* < 0.001.

**Figure S5.** Knockout of EphB1 receptors in Vglut2^+^ DH neurons augments MOR-mediated antinociception. **A** Western blot showing that the protein expression of EphB1 receptors in the lumbar spinal cord was inhibited by infection with AAV-Vglut2-Cre-EGFP in EphB1^fl/fl^ mice. Left, representative bands. Right, statistical summary. n = 3 in each group. **B**-**D** Knockout of EphB1 receptors in DH Vglut2^+^ neurons enhanced the analgesic effects of intrathecal administration of DAMGO (**B**), but not that of DPDPE (**C**) or U69593 (**D**), in the tail-flick test (left) and hot-plate test (right). Upper, %MPE. Lower, AUC. Sample sizes were indicated in brackets. Statistical analysis by unpaired Student *t* test in (A) to (D), or two-way ANOVA with Bonferroni’s multiple comparison test in (B) to (D). **P* < 0.05, ***P* < 0.01, and ****P* < 0.001.

**Figure S6.** STA-013 inhibits ephrinB2-Fc-induced internalization of MOR. **A** Immunofluorescence staining showing that STA-013 (50 μM) inhibited ephrinB2-Fc-induced internalization of MOR in Neuro-2a cells overexpressing MOR. Left, representative images. Scale bar indicates 10 μm. Right, statistical summary. n = 81 cells in IgG-Fc + Vehicle group, n = 70 cells in IgG-Fc + STA-013 group, n = 97 cells in ephrinB2-Fc + Vehicle group, and n = 121 cells in ephrinB2-Fc + STA-013 group. Statistical analysis by one-way ANOVA with Bonferroni’s multiple comparison test in (A). ****P* < 0.001.

**Figure S7.** Knockout of EphB1 receptors in Vglut2^+^ DH neurons inhibits morphine-induced phosphorylation of MOR at Ser^375^ in mouse models of pathological pain. **A**, **B** Western blot showing that knockout of EphB1 receptors in Vglut2^+^ neurons inhibited morphine-induced phosphorylation of MOR at Ser^375^ in DRGs (**A**) and the spinal cord (**B**) in mouse model of TCI-induced BCP. Left, representative bands. Right, statistical summary. n = 3 in each group. **C**, **D** Western blot showing that knockout of EphB1 receptors in Vglut2^+^ neurons inhibited morphine-induced phosphorylation of MOR at Ser^375^ in DRGs (**C**) and the spinal cord (**D**) in mouse model of PSNL-induced neuropathic pain. Left, representative bands. Right, statistical summary. n = 3 in each group. Statistical analysis by one-way ANOVA with Bonferroni’s multiple comparison test in (A) to (D). **P* < 0.05 and ***P* < 0.01.

**Figure S8.** EphB1 receptors interact with GRK3 in DRGs and the spinal cord. **A**, **B** Co-IP analysis showing the interaction between EphB1 receptors and GRK3 in DRGs (**A**) and the spinal cord (**B**) in native mice. DRGs or spinal cord lysate was immunoprecipitated with control IgG or EphB1 receptor antibody, and immunoblotted with antibodies against EphB1 receptor, GRK3, and GAPDH. DRGs or spinal cord samples were collected from three mice.

**Figure S9.** Cellular distribution of GRK2 in DRG and the spinal cord. **A** RNA scope assay and immunofluorescence staining showing colocalization of *Grk2* mRNA with CGRP, IB4, and NF200 in DRG neurons. Left, representative images. White arrows indicate double-labeled neurons. Scale bars indicated 50 μm. Right, statistical summary. 1137 CGRP^+^ neurons, 1394 IB4^+^ neurons, and 1775 NF200^+^ neurons from three mice were analyzed. **B** RNA scope assay showing the distribution of *Grk2* mRNA in Vglut2^+^ excitatory neurons (*Slc17a6*) or in Vgat^+^ inhibitory neurons (*Slc32a1*) in the DH. Left, representative images. White circles indicate double-labeled neurons. Scale bars indicated 50 μm (left) and 20 μm (right). Right, statistical summary. Nine spinal cord slices from three mice were analyzed.
